# Grandstanding Instead of Deliberative Policy-Making: Transitional Justice, Publicness and Parliamentary Questions in the Croatian Parliament

**DOI:** 10.1080/17502977.2024.2362001

**Published:** 2024-07-01

**Authors:** Denisa Kostovicova, Lanabi La Lova

**Affiliations:** European Institute, London School of Economics and Political Science, London, UK

**Keywords:** Transitional justice, publicness, deliberation, parliamentary questions, Croatia

## Abstract

Addressing the legacy of human rights violations in public can benefit victims, post-conflict societies and democracy building. But publicness of transitional justice (TJ) processes can also have opposite effects. We assess the relationship between publicness and TJ by leveraging the democratic deliberation theory concerned with the impact of publicness on the quality of policy-making. A comparative analysis of oral and written questions about TJ in the Croatian parliament (2004–20) shows that members of parliament use oral questions for nationalist grandstanding and written questions for substantive TJ policy deliberation. We demonstrate how publicness afforded by parliaments stymies TJ’s normative goals.

## Introduction

At the public commemoration ceremony honouring the victims of Chile’s military junta, the first post-dictatorship president, Patricio Alwyn, recited the names of the disappeared in a national public address. The ceremony took place in the National Stadium, which operated as a makeshift prison where some 20,000 men and women were tortured and some 40 murdered after General Augusto Pinochet seized power in Chile in 1973 (Waldstein 2015). As Alwyn recited the names, they appeared in bright letters on the stadium’s scoreboard ‘in a publication of retraction and apology to the victims of governmental wrongdoing’ (Teitel 2000, 126). During Pinochet’s nearly two-decade-long rule, the military regime carried out widespread repression, resulting in the disappearance, kidnapping, torture and killing of thousands. The public form of moral reparations for the victims, stigmatized as enemies of the state, was specifically recommended by the Chile’s Truth and Reconciliation Commission (TRC) (Bakiner 2015, 131–136; Teitel 2000, 126). This commemoration demonstrates how a transitional justice (TJ) instrument – in this case, symbolic reparations – depends on publicness, understood here as occurring in public, to achieve the aim of acknowledging the victims. But, there are also examples contrary to this, where the non-public nature of a TJ practice is essential for the recognition of harms. One such case is ensuring the confidentiality of testimonies from victims of gender-based wartime sexual violence when applying for reparations.

The relationship between publicness and TJ is complex and poorly understood in the extant scholarship. Publicness and TJ intersect in ways that either advance or undermine the aim of promoting reconciliation and restoring the dignity of the victims. In this article, we investigate how publicness affects TJ policy deliberation in parliament in a post-conflict society through the case of parliamentary questions (PQs). We analyze comparatively oral and written parliamentary questions about TJ asked by members of the Croatian parliament (MPs) from 2004 to 2020. The analysis brings together the scholarship on TJ and the theory of deliberative democracy, given their common interest in publicness and its effects on normative outcomes of justice and deliberative processes respectively.

Oral PQs sessions – often broadcast on TV, radio, and shared on social media – present a unique opportunity for members of parliament (MPs) to signal their worthiness as elected representatives (Martin 2011; Russo and Wiberg 2010; Saalfeld 2011). Politicians often use TJ issues to publicly boost their own nationalist credentials and subvert the aims of TJ (Loyle and Davenport 2016), for example, by acknowledging victims only from their own group. Publicity in policy deliberation has its ‘dark side’ (Chambers 2004, 389). Publicity or publicness can encourage grandstanding.^1^ It denotes a strategic use of speech to pander to the audience and enhance personal status and recognition at the expense of substantive policy deliberation (Park 2021, 216; Slapin et al. 2018; Tosi and Warmke 2020). We argue that publicness will have an adverse impact on policy deliberation about TJ in parliament, since oral questions will be used by MPs for their narrow personal and political gains.

Having compiled an original dataset comprised of oral and written PQs about TJ in the Croatian parliament from 2004 to 2020 and another dataset containing attributes of MPs and members of government in Croatia,^2^ we apply binomial modelling to examine the effect of publicness on TJ policy deliberation. Our findings show that oral questions about TJ are often employed as a tool for national(ist) grandstanding,^3^ evidenced by the MPs’ tendency to restrict the breadth of TJ-related questions to the most nationally salient aspects of TJ and to interact with co-partisans, i.e. government ministers from their own party or coalition partners. In contrast, written questions, which are not asked publicly, lend themselves to enhanced TJ policy deliberation because they allow for a more far-ranging discussion of TJ policy along with MPs’ more interactions across party lines as opposed with their own party members.

Our paper advances discussions about the role of publicness in TJ and contributes to its empirical evaluation. Our finding that publicness undermines TJ policy deliberation aligns with similar results on the drawbacks of transparency in parliamentary deliberations on other policy issues, where publicness reduces voicing dissent (Meade and Stasavage 2008), encourages more disrespectful discourse (Steiner et al. 2004) and where moral grandstanding leads to ideological polarization and political conflict (Grubbs et al. 2019; 2020). Our research on MPs in a post-conflict parliament offers a new perspective on how local actors stymie TJ. We echo the argument by Gready and Robins (2014) that local actors – in our case, MPs – should not be romanticized. We advance the argument to show under what conditions MPs would be likely to obstruct TJ. Lastly, by using new forms of data, namely, digitized Croatian PQs, our research takes advantage of technological advancement to diversify the evidence base for normative claims in TJ (Pham and Aronson 2019).

## Publicness and transitional justice: A complex relationship

Publicness, understood as taking place in public, as opposed to being secret or private, serves an important role in addressing the legacy of mass atrocity and human rights violations. As Dempster (2020, 250) argues, publicness is not ‘a technical matter’, but a fundamental TJ principle that facilitates profound transformation of post-conflict societies. Moral benefits of publicness to victims and societies at large, as well as political benefits of publicness for democracy and peacebuilding, have been theorized. Yet, these benefits for victims, societies and democracy are not ubiquitous. There are situations where the lack of publicness is needed to reach TJ’s normative aspirations both for individuals and societies.

Public acknowledgment of responsibility for wrongdoing is critical for restoring the dignity of victims (Dempster 2020, 248–250; Kirk 2016). It serves as ‘a powerful tool in effecting healing’ of people affected by violence and of societies at large (Sooka 2006, 319). It also has a potential to mobilize victims’ participation in TJ and their willingness to testify (Winston 2021). Moral rejection of human rights violations in public has political implications. It delegitimizes the previous regime along with its abuses (Teitel 2000, 5) and promotes more local engagement, activism (Dancy and Thoms 2022) and democratization (Taylor and Dukalskis 2012), for example, through modelling procedural fairness in the operation of TJ (Gibson 2009). Additionally, publicness can have an indirect social impact. Although the South African TRC has been criticized for instituting the amnesty of perpetrators, the public nature of amnesties has led to their shaming, ultimately reducing perpetrators’ power and ability to reoffend (Van Zyl 1999, 661–662). Similarly, limiting the public nature of inquiries can undermine their truth-finding purpose because it impedes persuading the public of the veracity of the findings. This was the case with the Bloody Sunday inquiry, where public access was curtailed due to invoked national security concerns (Hegarty 2002, 1176–1177).

However, publicness can also stymie TJ, adversely affecting victims, societies and broader political aims, such as democratization. Public apologies can neglect victims because these apologies are performative and monologic in nature, whereas ‘forgiveness is predicated’ on an interpersonal and dialogic interaction (Espindola 2013, 328). Alternatively, testimonies of victims of wartime sexual violence need to be out of the public eye to ensure their participation in TJ processes (Okello and Hovil 2007). Operating as ‘public spectacles’, TRCs can draw attention from other significant issues, such as corruption, and hinder institutional reforms (Dancy and Thoms 2022, 565). Also, negotiations behind the scenes can facilitate the development of TJ legislation (Dempster 2020). Elsewhere, blurring the private and public nature of TJ enables reckoning with past wrongs. This is illustrated by an Albanian mother turning her home into a museum to commemorate the deaths of her sons in the Kosovo War (Schwander-Sievers and Klinkner 2019).

Practitioners have considered the effects of publicness. For instance, Peru’s TRC chose not to allow public hearings of over 1,000 rebels it interviewed because of the concern that their testimonies might undermine the Commission’s goals (O’Connell 2021, 154–155). But, by and large, practitioners have focused on public outreach because involving the public has been critical to designing a legitimate TJ approach that resonates with local needs. Likewise, public education through impartial information is key to creating confidence in the operation of TJ instruments and their findings (Lincoln 2011). This has been critical for tackling misrepresentation of TJ practices, driven strategically by political elites to secure impunity or advance other narrow political ends (Hehir 2019), although outreach itself is not immune to co-option by actors disinterested in justice (Lambourne 2009; Salehi 2023).

In the extant literature, the terms ‘publicness’ and ‘public’ have been used to signify the public nature – in terms of being public rather than secret or private – of a whole range of referents: actors, movements, processes, events, documents or other phenomena, such as knowledge. Considering the breadth of referents, along with varied effects of publicness on TJ goals, comparison across cases can only be made at a stretch. To advance the understanding of the relationship between publicness and TJ, we need to be explicit about the meaning of publicness in the context of research. For example, Dempster (2020) defines publicness as constituted by three elements: performance, audience and collective history-making. Alternatively, Holder (2017) questions who constitutes the public. We leverage the democratic deliberation theory to study publicness and TJ. Drawing on this ‘talk-centric’ theory, we specify the meaning of publicness as public policy deliberation (Bächtiger et al. 2005, 225), which we operationalize through a comparison of oral and written parliamentary questions about TJ.

## Policy deliberation: Benefits and drawbacks of publicity

The theory of democratic deliberation focuses attention on how people formulate arguments when addressing political problems in public. Thompson (2008, 498) specifies that ‘[c]itizens and their representatives are expected to justify the laws they would impose on one another by giving reasons for their political claims and responding to others’ reasons in return’. In addition to rational justification and reciprocity, deliberation also requires civility, equality in participation and consideration of the common good in formulating political claims (Habermas [1984] 2004; Gutmann and Thompson 1996; Steiner et al. 2004). These normative requirements of deliberative discourse have guided the empirical study of deliberation and measurement of the quality of discourse during deliberation processes and its effects relevant to policy-making, such as the legitimacy of political decisions (Bächtiger and Parkinson 2019). In these efforts, publicness – considered a defining characteristic of democratic deliberation – has been shown to have ambiguous effects.

Theories of democratic deliberation associate publicity with ‘salutary’ effects (Chambers 2004). Publicity pushes deliberators away from self-interest (for example, ethnic interests) towards consideration of the common good (Chambers 2004, 390–391). Because deliberators focus their minds on presenting arguments and considering counterarguments, publicity provides transparency to the decision-making process (Karpowitz and Raphael 2014).^4^ Acknowledging the necessity of publicity for democratic deliberation, Hayward (2021, 179) notes its importance for ensuring openness, inclusivity and politicization of issues, which ‘encourage[s] people to understand themselves as political actors who care about public things’, and motivates them ‘to participate in caring *for* those public things’. Furthermore, according to Hayward (2021, 176), publicity matters because it ‘constrains and enables people to manage their relations democratically’. However, other scholars criticize ‘taken-for-grantedness’ of publicity in the context of democratic politics and, specifically, democratic deliberation (Dean 2001, 625).

Publicity has its ‘dark side’, whose ‘harmful effects […] have been under-theorized’ (Chambers 2004, 389). Scholars have argued that deliberating away from the public eye can be more conducive to formulating public reason. Elster ([1986] 2010), one of the most vocal proponents of secrecy as opposed to publicity, has argued that publicity has a negative effect on the quality of discourse. Similarly, as Chambers (2005, 259) pointed out, even the staunchest proponents of publicity, Gutmann and Thompson (1996), accept that deliberative secrecy is conducive to more candid and thorough consideration of issues and, consequently, can motivate a change of opinion – a key benefit of good-quality deliberation.

These theoretical insights about contradictory effects of publicness on policy deliberation have been backed with empirical evidence. Findings point to the decrease in the quality of deliberation in face-to-face discussions versus closed settings in parliaments (such as committees) (Meade and Stasavage 2008; Steiner et al. 2004) and highlight the low quality of deliberation in the context of technologically mediated, hyper-public social media environments (Quintero Ramírez 2021, 26). Question-asking is also a form of deliberation (Ilie 2022). As such, questions are an important indicator of deliberative quality (Rowe 2015, 549). The democratic deliberation perspective applied to TJ centers on the quality of policy deliberation about TJ evidenced in the case of PQs, which will be impacted by publicity. This, in turn, will have second-order effects on a range of outcomes concerning victims and societies, along with democracy building and peacebuilding.

## (Ab)uses of parliamentary questions about transitional justice: Hypotheses

Human rights violations committed either through conflict or regime repression put TJ issues on the political agenda. The context in which deliberation unfolds is relevant for the quality of public debates (Ruiz et al. 2011, 482). Post-conflict societies present unique challenges for the prospects of policy deliberation. TJ is conducive to being abused because of the political environment, defined by mistrust and enduring polarization of communities. Nonetheless, scholars, albeit primarily through the study of small group communication, have demonstrated the positive effects of deliberation in divided societies. One of these is encouraging reconciliatory attitudes (Luskin et al. 2014; Steiner 2012; Ugarriza and Trujillo-Orrego 2020).^5^ Post-conflict parliaments present another arena where deliberation takes place ‘under the “glare” of publicity’ (Chambers 2004, 389), with a potentially adverse impact on TJ deliberation.

Post-conflict publics are typically socialized into ethnocentric attitudes on conflict-related issues, such as TJ (Gordy 2013). At the same time, war discourses and narratives persist in parliamentary debates long after a conflict is over (Mochtak 2020; Mochtak, Glaurdic, and Lesschaeve 2022). As an issue related to conflict, TJ holds national resonance and acts as a symbolic axis for the articulation of national identity in post-conflict societies (Russell-Omaljev 2016). Therefore, addressing the issue of TJ publicly can provide a unique opportunity for MPs to enhance their political standing by gaining visibility. For this reason, MPs may choose the oral form of a PQ about TJ over the written one to enhance their national standing (Bailer 2014). Taking these considerations into account, we formulate our first hypothesis.

*H1: Questions related to TJ, in comparison to all the other questions asked in the parliament, are more likely to be oral than written*.

While TJ holds paramount symbolic importance to nations emerging from conflict, it is typically a vehemently contested issue. The articulation of national identity through TJ pits nationalist political forces against liberal ones, as manifested in public discourse (Russell-Omaljev 2016). Nationalist discourses contest or deny ingroup responsibility for war crimes, while exclusively focusing on their own victims (Cohen 2013). Unlike them, more moderate and liberal voices align with the global norm of accountability, demanding responsibility for all war crimes and acknowledgement of all victims regardless of their identity (Bešić and Džuverović 2020). PQs provide a platform for public contestation along nationalist–liberal lines in a post-conflict society. They allow MPs to make a public stance on a particular issue and cultivate the relationship with their constituencies. Scholars have shown that considerations of identity can serve as a powerful motive for asking questions. For example, MPs can use PQs to cultivate connections with immigrant and minority communities by asking questions related to their concerns (Saalfeld 2011). Similarly, female legislators ask more questions about issues that concern and affect women (Bäck, Debus, and Müller 2014; Jacob 2014, 253–254; Mügge, van der Pas, and van de Wardt 2019). Hence, consideration of an issue salient to national identity can be an equally powerful motive for asking a related parliamentary question. Nationalist politicians have adeptly exploited TJ to boost their nationalist credentials outside the parliamentary chamber (Subotić 2009). Considering that oral questions draw media attention and generate personal publicity and benefits to MPs (Norton 1993; Wiberg and Koura 1994, 30–31), we hypothesize that right-wing MPs will be more likely to exploit oral PQs about TJ to ‘mark their territory’ (Guinaudeau and Costa 2022, 511) and enhance their nationalist credentials. But, for moderate and liberal politicians, taking a critical stance publicly toward their nation’s wrongdoing will be costly since they risk being ostracized as traitors (Russell-Omaljev 2016). This may deter them from asking oral PQs about TJ.

*H2: Oral questions related to TJ, in comparison to written questions about TJ, are more likely to be asked by politicians from nationalist parties*.

The interactions between MPs and government ministers serve various purposes; even a single question may have multiple functions (Rozenberg and Martin 2011). PQs are an important tool for constituency representation and gathering personal votes (Martin 2011; Russo and Wiberg 2010; Saalfeld 2011). However, they can also generate major benefits for the party of an MP who is asking the question. Oral questions tend to focus on topical policy issues and serve more to criticize or praise ministers than to obtain either hidden or concrete information (Rozenberg and Martin 2011). PQs are also used effectively as ‘a tool of partisan differentiation’ (Guinaudeau and Costa 2022, 519) and serve as an integral part of party competition (Eissler et al. 2023, 360; Otjes and Louwerse 2018). An oral question addressed to a co-partisan minister gives a minister an opportunity to showcase their party’s record or position on a policy issue. For example, the questions addressed to co-partisans tend to carry a more positive sentiment compared to those directed to ministers from other (opposition) parties (Kukec 2022). At the same time, PQs are used to ‘politicize issues as they increase their salience and express partisan divergences’ (Guinaudeau and Costa 2022, 511). However, benefits from politicizing TJ in a post-conflict context are uncertain. MPs may lose out if they cannot control the narrative on TJ – a nationally contested issue – when directing a parliamentary question to political opponents. Therefore, we hypothesize that it will be politically ‘safer’ for MPs to direct an oral question about TJ to a co-partisan member of the executive, thereby providing them with a public platform to praise or advocate their stance on the issue, while using written questions out of the public eye for partisan interactions.

*H3: Oral questions related to TJ, in comparison to written questions about TJ, are more likely to be addressed to co-partisans*.

TJ as a policy issue has various dimensions. For example, different aspects of a TRC need to be disaggregated to gain better understanding of its effects, which can include fact-finding, deterrence or creation of a historical record, among others (Wilson 2001). Similarly, deliberations about TJ within a peace process or in a national parliament encompass its different aspects, some of which carry greater national symbolism than others. For example, the issue concerning who is entitled to reparations when ethnicity plays a role is more sensitive than the one concerning a media strategy on TJ reporting (although both are contentious and highly likely to be politicized in post-conflict societies). PQs can encourage the consideration of a range of possible actions as propositions to be evaluated before making a decision (Snedegar 2019, 688). The same applies to TJ, which represents a multifaceted policy. Given that the most nationally salient issues are linked to the greatest personal benefits from grandstanding (Park 2017), we can expect that the MPs will focus on those most nationally salient dimensions of TJ policy when asking oral PQs about TJ. In contrast, written questions are less public. While these PQs can still be accessed publicly, they are not disseminated as widely (Rozenberg and Martin 2011). Importantly, written PQs are also relatively unconstrained because MPs can represent their constituents without being limited by partisan issues (Saalfeld 2011). Oral questions also tend to be less substantive than written ones, in which ‘ministers are asked to give precise, reliable and opposable information about their past activities or their future plans’ (Rozenberg and Martin 2011, 395). Because written questions are less public, we hypothesize that MPs will have no incentive to use written PQs to address the most nationally salient aspects of TJ, which, in turn, will promote TJ policy deliberation.

*H4: Within TJ as a policy issue, oral questions, in comparison to written questions, are more likely to concern its most nationally salient aspects*.

## Parliament, politics and transitional justice in post-conflict Croatia

### War and TJ in Croatia

The Croat–Serb war on Croatia’s territory that took place from 1991 to 1995 was triggered by secessionist claims by Croatia’s ethnic Serb minority, which were supported by neighboring Serbia. As neighbors became enemies and turned against each other (Dragojević 2019), many civilians were killed, expelled from their homes and brutalized in detention camps, run not only by Serbs but also by Croats. In a major military operation, the Croats expelled most of the Serb population from Croatia before the end of the war (Tsai 2021).

After the signing of the 1995 Erdut peace agreement, the war was portrayed in Croatia as an existential fight for the nation’s survival. This idea was reflected in the official label for the war: ‘the Homeland War’. The Croatian political leadership crafted the national narrative of the Croat–Serb conflict as a defensive and just war. Such a conception resonated among a broad section of the Croatian society (Pavlaković 2010; Sokolić 2019). This hegemonic national narrative of the Croat–Serb war framed post-conflict TJ efforts. Foremost attention was given to ethnic Croat victims, overlooking ethnic Serb victims of violence. The responsibility for war crimes committed by ethnic Croats was contested (Ljubojević 2012). Prioritizing recognition and compensation of Croat war veterans marginalized the needs of other Croat and non-Croat civilian victim groups, for example, female victims of sexual and gender-based violence.

The ethnocentric national narrative of war and victimhood has remained largely unchanged over time. Croatia’s politicians used the work of the International Criminal Tribunal for the Former Yugoslavia in the Hague (ICTY), where Croats were suspected of war crimes (along with members of other ethnic groups involved in the former Yugoslavia’s violent dissolution), to assert the nation’s victimhood, while contesting Croats’ responsibility for war crimes committed against ethnic Serbs (Grodsky 2009; Subotić 2009). Similarly, domestic war crimes trials in Croatia have targeted ethnic Serbs’ perpetrators (Vajda 2019). Isolated instances of acknowledging victims from other ethnic groups have been unable to shift the dominant nationalist discourse and narratives centred on TJ (Banjeglav 2012).

In post-conflict Croatia, TJ became a highly politicized issue of national importance, delineated by normative boundaries of the acceptable ethnocentric conception of justice. Politicians used TJ to discredit opponents and assert their nationalist credentials. Any criticism of Croatia’s nationalism, as reflected in TJ debates, was portrayed as a betrayal of the nation. National policy deliberations about TJ, including those in the Croatian parliament, have taken place in the context of an emerging multi-party democracy.

### Nation-building and party formation in Croatia

Croatia is a multi-party democracy that joined the European Union in 2013. The Croat–Serb war led to ‘ethnification’ of politics that endured beyond the end of the conflict (Dragojević 2019; Kasapović 1996). The ethnification process rests on framing political issues in ethnic terms, creating a rift between those perceived to be committed to the nation and others seen as betraying it. These dynamics have underpinned the formation of Croatia’s multi-party system, where party politics is characterized by competition between the two main parties. The Croatian Democratic Union (HDZ), whose founder and national leader Franjo Tudjman led the war effort in Croatia, is right-of-centre, whereas the Social Democratic Party (SDP) is left-of-centre. According to Dolenec (2012), although socioeconomic cleavages exist in the Croatian society, they do not form the basis for political party competition. Instead, Croatia’s parties are steeped in ethnic politics, with ethnic outbidding as a mode of political competition spearheaded by the HDZ (Marijan 2018).

**Figure 1.**
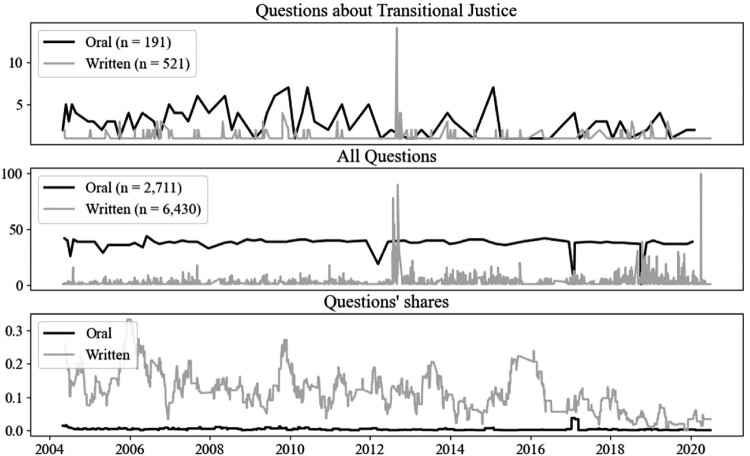
Daily counts of questions about TJ and all questions in the parliament.

The two parties participated in the first democratic elections in 1990 (Čakar and Čular 2016).^6^ The parties symbolize two faces of Croatian nationalism, with the HDZ and the SDP representing its more ethnic and more civic faces, respectively. However, it is harder to draw the distinction between the two parties in relation to the Croat–Serb war. The liberal SDP voted in favour of the Declaration on the Homeland War in 2000, the document that defined the war as defensive, legitimate and just (Jović 2017). Although this Declaration narrowed the political space for a critical approach to accountability for crimes committed by Croats during the conflict, the SDP did support Croatia’s collaboration with the ICTY and accountability for Croats’ responsibility for wartime wrongdoing. Nearly three decades after the end of the Croat–Serb war, the discourse of war is still prominent in the broader political environment (Sokolić 2019) and Parliament (Mochtak 2020; Mochtak, Glaurdic, and Lesschaeve 2022). Similarly, parliamentary questions about TJ, as a conflict-related issue, have figured steadily during the Question Time in parliament, as shown in Figure 1.

## Data and methods

We analyze the original data set comprising 712 (191 oral and 521 written) PQs about TJ asked in the Croatian parliament from 2004 to 2020. The questions were sourced from a larger dataset of all the oral (2,711) and written (6,430) questions downloaded from the website of the Croatian parliament called *Sabor*. We extracted the questions applying a problem specific dictionary of search terms (Rooduijn and Pauwels 2011), using both deductive and inductive approaches (Neuendorf 2002, 126–130). The deductive approach encompassed TJ terms used universally (such as war crimes, victims, transitional justice, truth commission, memorialization, etc.), whereas the inductive approach included TJ terms specifically related to Croatia (such as local justice initiatives; domestic trials; ICTY; the names of individuals indicted by the ICTY, such as Norac, Praljak, Gotovina and others).

Like in other parliaments, asking oral and written questions is a regulated parliamentary activity (Poslovnik Hrvatskog Sabora 2020). Oral questions are asked during the Question Time (Aktualna prijepodneva, n/d)^7^ at the beginning of each parliament session, following rules that ensure equal opportunities for MPs to ask a question (Kukec 2022; Poljak 2022a; Poljak 2022b). Written questions are handled by the Parliament Speaker, who directs the question to members of the government. The Question Time in the Croatian parliament attracts media attention (Arapović and Špoljar 2023). Reports on oral PQs, especially if they are contentious, appear in Croatia’s press, are broadcast on TV and are shared on social media.

To date, scholars have analyzed oral PQs in Croatia to gain insight into attacks and incivility in the parliament (Poljak 2022a; 2022b) and the role of partisanship (Kukec 2022). Our corpus that consists both of oral and written PQs provides a valuable source of data to evaluate the impact of publicness on TJ policy deliberation by conducting their comparative analysis. For our analyses, we compiled two datasets.

One dataset comprises the metadata related to all oral and written PQs asked in the period from 2004 to 2020 (9,141 questions, 712 of which are about TJ). For each entry, it provides the name and party membership of the asker and the answerer, the policy area of each question and the date when the question was asked.

Another dataset comprises the attributes of all MPs and government ministers in the Croatian parliament in the same period. We coded manually a range of individual-level characteristics for 777 MPs and the members of the executive involved in PQs and answers. These include the information on sociodemographic attributes (such as age, gender and education), party membership, coalition membership, party’s ideology,^8^ government/opposition membership and the severity of conflict impact on electoral units.^9^

To identify the differences between the asking of oral and written questions, we estimate the coefficients for multiple specifications of binomial regressions, with the outcome variable that equals one if the question is oral (and zero otherwise) and individual-level characteristics of the questions, MPs and members of the executive involved in the questions and answers as explanatory variables. Our tests for *H1* rely on the analysis of the metadata related to all (oral and written) questions, whereas tests for *H2–H4* draw on the subset of oral and written questions that relate to TJ.

## Results and analysis

To evaluate the effect of publicness on TJ policy deliberation, we first hypothesized that TJ, being an issue of national importance, may be used to assert national standing and, therefore, may more likely be addressed in oral questions (as formulated in *H1*). Our results suggest that even though TJ was a relatively popular policy issue that MPs referred to in their oral and written PQs (Figure 1), the questions about TJ were not more likely to be asked orally than in the written form, compared to all the other questions on other policy areas in the Croatian parliament from 2004 to 2020 (Table 1). This finding indicates that, although TJ as a policy issue presents an opportunity for MPs to exploit publicness for personal ends and enhance their national standing, MPs seem to restrain themselves from using it; when asking a question about TJ, they are more likely to stick to the written form.

**Table 1.** All oral questions, correlates: binomial regression results.

	(1)	(2)	(3)
TJ	−0.151* (0.088)	**−0**.367*** (0.102)	**−0**.716*** (0.110)
Within party		2.795*** (0.079)	2.662*** (0.091)
Party ideology of asking MP			
far-right			0.725*** (0.175)
right-of-centre			1.015*** (0.092)
left-of-centre			0.178** (0.080)
NA (independent)			0.815*** (0.131)
Controls			yes
Intercept	−0.852*** (0.024)	−1.268*** (0.028)	−0.493 (0.450)
Observations	9,142	9,142	9,142
McFadden’s pseudo-Rsq	0.0003	0.1537	0.2558

Note*:* The dependent variable equals one if the question is oral and zero if the question is written. Standard errors in parentheses. ***p ≤ 0.001, *p ≤ 0.01. Controls include categorical variables for the parliamentary terms, gender of the MPs and cabinet members, age and education of the MPs. Independent (non-partial) MPs asked 6 per cent of all the questions. See A6 in the Online Appendix for the estimates of the coefficients for controls in Model 3. ‘Centre’ (for instance, HSLS – Croatian Social Liberal Party) is taken as a base party ideology category.

TJ is a contested topic in Croatia (Pavlaković 2010; Sokolić 2019). Therefore, asking a question about TJ may easily backfire, especially if an MP dissents from the widely accepted ethnocentric conception of TJ in Croatia. This is evident in Table 1, which reports the coefficients for three specifications of binomial models, with the dependent variable being equal to one if the question is oral. The coefficient for a binary variable TJ (equals one if the question is about TJ) is always negative; the result is robust across various specifications and significant at 0.01 level. A question about TJ, as opposed to a question about any other topic, is less likely to be asked orally. Table 1 demonstrates that the logarithm of the odds of a question about TJ being asked orally varies from −0.151 to −0.716, depending on the model’s specification. For instance, based on the results from Model 1, a predicted probability of a question being asked orally, given it covers the issues of TJ, is only 0.268.

The direction of the relationship revealed by our tests of *H1* – that is, a negative and statistically significant coefficient for the variable TJ – is the same in more complex models, e.g. Models 2 and 3, that include further controls (or combinations of controls), such as gender of the asker and their executive target, their education, age, government/opposition membership, parliamentary term, ideology of the asker and the answerer, and the severity of conflict impact on the electoral unit. All the additional controls that we included in our tests, except for the variable that measures the severity of conflict impact, displayed a statistically significant link with the outcome. Moreover, in models with additional controls, the absolute value of the estimate of the log odds of a question being asked orally is higher in magnitude, which provides additional evidence against *H1* and in favour of our findings*.* A typical example of a specification that includes controls is provided in Model 3 (Table 1).

We now turn to *H2*–*H4,* which are concerned with the differences between oral and written questions about TJ and, therefore, were tested exclusively on the dataset comprised of the questions about TJ. Our results demonstrate strong evidence in favour of *H2*–*H4* (Table 2 provides selected results of the regressions), which test whether an oral PQ will be asked by a nationalist, target a co-partisan and concern the most nationally salient aspect of TJ. The direction of the estimates that refer to *H2*–*H4*, which we present in Table 2, consistently holds in more complex models that include variables and the combinations of variables for the ruling coalition membership, gender, education, age, cabinet membership, parliamentary term, party ideology of individuals involved in the PQs and the severity of conflict impact on the electoral unit.

**Table 2.** Oral questions about TJ, correlates: binomial regression results.

	(4)	(5)	(6)	(7)
Co-partisan	0.543*** (0.045)	2.948*** (0.313)	2.900*** (0.314)	1.858*** (0.521)
Party ideology of asking MP				
far-right	0.165** (0.081)	1.554*** (0.561)	1.502*** (0.563)	1.669*** (0.571)
right-of-centre	0.136*** (0.048)	1.033** (0.399)	0.953** (0.401)	0.968** (0.403)
left-of-centre	0.063 (0.04)	0.886*** (0.37)	0.826** (0.372)	1.000** (0.385)
NA (independent)	0.106 (0.083)	1.395** (0.619)	1.374** (0.618)	1.548** (0.625)
Within the ruling coalition				1.212** (0.491)
Veterans and Homeland War policy area			0.376* (0.216)	0.421* (0.219)
Controls	no	yes	yes	yes
Intercept	0.093** (0.034)	−3.180*** (0.776)	−3.309*** (0.778)	−3.524*** (0.800)
Observations	712	712	712	712
McFadden’s pseudo-Rsq	0.2633	0.2709	0.2135	0.2565

Note*:* Dependent variable equals one if the question is oral and zero if the question is written. Standard errors in parentheses. ***p ≤ 0.001, **p ≤ 0.05, *p ≤ 0.01. Controls include categorical variables for gender, age, education and parliamentary terms. Independent (non-partial) MPs asked 4 per cent of the questions about TJ. ‘Centre’ is taken as a base party ideology category.

**Figure 2.**
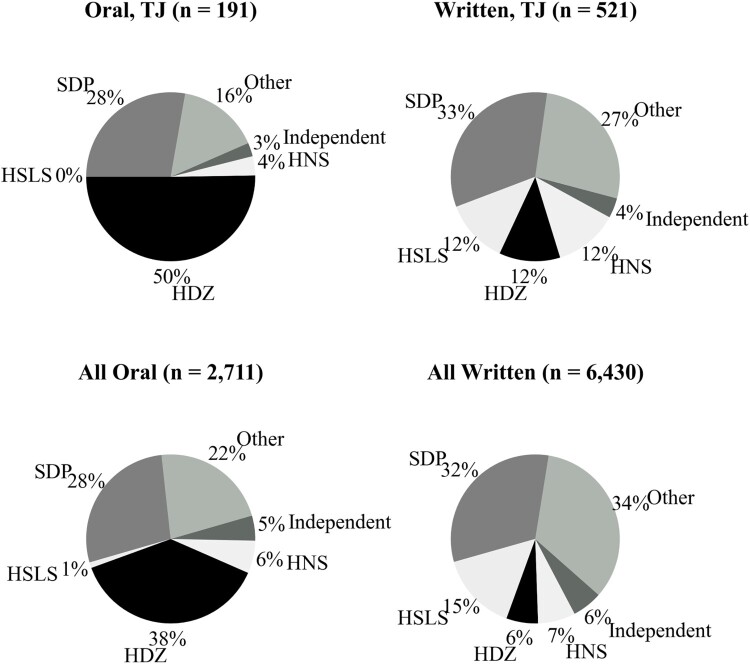
Party of an asking MP. Note: HSLS stands for the Croatian Social Liberal Party and HNS for the Croatian People’s Party – Liberal Democrats. The percentages presented in the upper left pie chart exceed 100% due to rounding.

We find strong evidence in support of *H2*: oral questions about TJ, in comparison to written questions, are more likely to be asked by the politicians from nationalist parties (Figure 2), holding other variables constant (Table 2, Models 4–7). For instance, based on Model 4, a predicted probability of a question being asked orally, given it is about TJ and is addressed to a member of a right-of-centre party by a co-partisan, is 0.772.

The choice between written and oral form varied significantly depending on an MP’s party. Descriptive evidence presented in Figure 2 illustrates that the members of HDZ asked most of the oral questions about TJ (96 questions, 50 per cent). However, HDZ (classified as right-of-centre) was markedly less active in asking written questions, having asked only 61 (12 per cent). In contrast, SPD (left-of-centre) follows, having asked approximately one third of oral PQs (53 or 28 per cent) and one third of written PQs (172 or 33 per cent).

The descriptive statistics presented in Figure 2 provide the breakdown of questions by the asking MPs’ party. HDZ uses the parliament’s public arena to ask disproportionately more oral questions about TJ in comparison to questions on other topics. Arguably, HDZ uses publicness strategically to showcase its nationalist credentials. However, legislators from more moderate parties, such as SDP or HNS, which have been historically vulnerable on the sensitive TJ issue, ask proportionally more questions about TJ in the written form. Being out of the public eye gives them a political space to probe TJ policy without being exposed to the punishment of not appearing sufficiently nationalist. Importantly, as we demonstrate in the Appendix, the observed results are unlikely to be due to the fixed quota related to the party size.^10^

**Figure 3.**
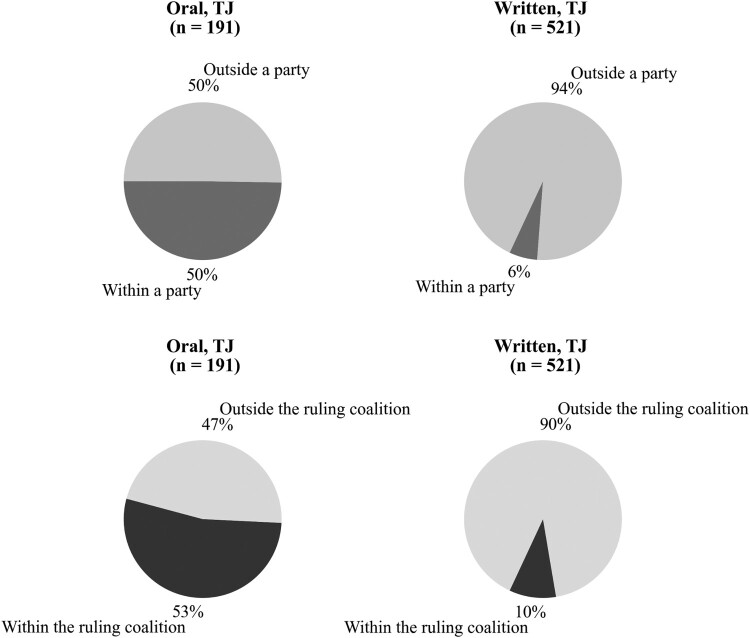
Questions by membership in the same party and in the ruling coalition (co-partisan and partisan interactions).

We also find evidence in support of *H3*: oral questions about TJ, in comparison to written questions, are more likely to be addressed to the government members of the same party, i.e. co-partisans, holding all the variables constant (Table 2). For example, based on Model 4, a predicted probability of a question being asked orally, given it covers the issues of TJ and is asked to a co-partisan by an MP from a right-of-centre party, is 0.773. This confirms Kukec’s (2022) finding on co-partisan patterns that refer to all oral questions. However, most of written questions about TJ, as opposed to about half of oral questions, are asked outside the party or a coalition, i.e. involve partisans (see Figure 3).

Our findings demonstrate that MPs use oral questions to showcase the interest of their party or of their party’s coalition partner in prominent national issues that concern TJ, while shying away from engaging political opponents. Hence, publicness encourages MPs to play safe. Addressing a PQ to a partisan and challenging TJ policy could facilitate policy deliberation. However, our findings suggest that MPs tend to avoid any risks inherent in deliberation of a highly sensitive TJ issue across the partisan divide, for example, the risk of them being punished for challenging the dominant nationalist narrative of TJ. This is evident if we consider that more than 60 per cent of oral questions addressed to co-partisans were asked about the policy area related to Croatian war veterans and the ‘Homeland War’, whereas only one third of written TJ questions addressed to co-partisans concerned these dimensions. Given the standing of war veterans as an especially deserving category for compensation and their significant role in the construction of the nationalist narrative of Croatia’s war (Sokolić 2019), the result indicates that oral questions are deftly used to signal concern for war veterans and enhance an asking MPs’ national standing, while giving the platform to her or his party to showcase that the party’s position on this issue resonates with the public.

**Figure 4.**
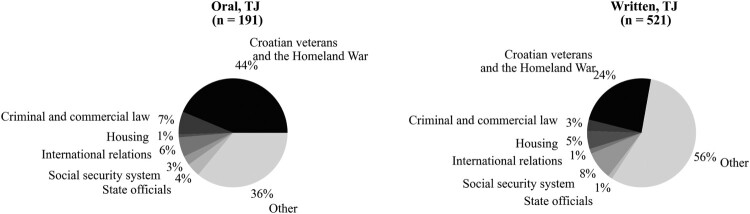
Policy areas within which the questions were asked.

Finally, we find evidence in favour of *H4*: oral questions related to TJ, in comparison to written ones, are more likely to address the most nationally salient aspects of the TJ policy (Model 6 in Table 2). Figure 4 shows that while 44 per cent of oral questions were asked in relation to Croatian war veterans and the ‘Homeland War’, only 24 per cent of written questions were asked in relation to these policy dimensions.^11^ This indicates that publicness reduces the potential diversity of questions related to TJ. In contrast, written questions are more conducive to diversifying TJ policy deliberation and encompass more aspects of TJ.

## Conclusion

We have analyzed the impact of publicness on TJ by focusing on TJ policy deliberation using the case of oral and written PQs. While transparency and openness are key requirements of democratic politics, MPs can also ‘turn one’s contribution to public discourse into a vanity project’ (Tosi and Warmke 2016, 199). Yet, publicness makes MPs vulnerable to political punishment and acts as a disincentive for criticism and dissent related to nationally contentious issues, such as TJ. By bringing the scholarship on TJ into dialogue with the democratic deliberation theory, we proposed and demonstrated that publicness encourages nationalist grandstanding in parliaments. MPs are inclined to use the opportunity afforded by oral PQs about TJ to assert and signal their nationalist credentials, undermining the normative goals of TJ.

Our findings demonstrate that oral PQs, unlike written ones, restrict TJ policy deliberation. Oral questions about TJ, as demonstrated in Croatia’s case, serve as a political resource for MPs from nationalist parties to dominate TJ policy deliberation. Further, by prioritizing recognition of war veteran-related issues over others, oral PQs narrow down the range of TJ’s different aspects that are discussed publicly, including those affecting civilian or female victims. In this way, oral questions serve a ‘predetermined agenda’ (Chambers 2005, 262; cf. Penner, Blidook, and Soroka 2006), which aligns with an ethnocentric and gendered TJ in Croatia. Conversely, as our findings show, written questions, tabled out of the public eye, are associated with substantive diversification of TJ policy deliberation, evidenced with more partisan interactions and engaging more with different aspects of a TJ policy. Scholars have argued that the publicness of oral PQs undermines their prospective contribution to ‘the understanding of politics and specific policy issues among the citizens as the primary audience of this event’ (Kukec 2022, 16; Rozenberg and Martin 2011). Our study reinforces this point through a comparison of oral and written questions about TJ, although the scope of our operationalization of the quality of TJ policy deliberation as the prevalence of partisan versus co-partisan interactions and the diversity of TJ as a policy needs to be noted.

We show that oral and written PQs provide an instructive case of TJ policy deliberation in parliament under different conditions of publicness. Applying quantitative analysis to new data availed by technological advancements, we contribute to growing evidence underpinning claims about the effects of TJ in post-conflict societies. Our study challenges the assertion that the application of quantitative methods in TJ contributes ‘to a form of decontestation’ of TJ (Lühe 2023, 1881). Quantitative analysis leads us to the discovery of a new way in which local actors, in this case MPs, politicize TJ to advance their narrow political ends. This, in turn, problematizes our conception of TJ in terms of what it is, who it is for and what it can do in post-conflict contexts. Nonetheless, considering the scope of our research, further insights can be gained both through quantitative and qualitative content analysis, such as Foucauldian discourse analysis or linguistic analysis of language, to draw out further differences of MPs’ public and non-public discourse as well as to capture the substantive depth of deliberation (Bächtiger and Parkinson 2019; Ilie 2022; Vliegenthart and Walgrave 2011). Even as it stands, the politicization of TJ in a national parliament, which we demonstrate with the case of PQs, presents a serious dilemma for scholars and practitioners interested in promoting justice.

We know that multiple goals of TJ may be at odds with each other (Dancy et al. 2019). Similarly, multiple mechanisms through which TJ operates, such as publicness, can have varied and contradictory effects. Publicness of TJ policy deliberation in a national parliament makes the process vulnerable to political exploitation, with multiple negative effects for victims, societies and democracy building. Oral PQs present an opportunity to publicly argue for recognition of all victims, but they also incentivize nationalist grandstanding and constructing hierarchies of victims that acknowledge some but not all victims. This tension undermines the prospects for justice. Our findings raise a difficult question about how parliaments in post-conflict societies can facilitate the kind of TJ policy deliberation that will promote justice and recognition of all victims.

## Supplementary Material

Supplementary Material

## Notes

1 The scholarship on deliberative democracy refers to the problem of publicness as that of publicity. Both terms address the implications of a public process, such as public policy deliberation (Chambers 2004, 391).

2 This covers the period from the 4th to the 9th term in the Croatian parliament. As the 4th term started on 22 December 2003, the PQs were effectively asked from the beginning of 2004.

3 We use nationalist grandstanding as a form of moral grandstanding, defined as ‘a use of moral talk that attempts to get others to make certain desired judgments about oneself’ (Tosi and Warmke 2016, 199). We draw attention to how speakers make use of issues related to the sense of national identity, as framed in the dominant nationalist discourse to achieve self-promotion.

4 Chambers (2004, 391) specifies one meaning of publicity: as policy deliberation taking place in public.

5 These deliberative mini-publics have been assembled in line with an experimental method or were a part of a deliberative poll, another method applied in the study of deliberation.

6 The HDZ won and remained unseated for a decade.

7
https://www.sabor.hr/hr/sjednice/aktualna-prijepodneva

8 This is based on the Chapel Hill Expert Surveys that estimate party positioning on ideology, available at: https://www.chesdata.eu/

9 There is no complete record of war dead in the Croat–Serb war. Our coding of electoral units relied on official records of the number of killed veterans (military) (Živić 2005a; 2005b), as well as on separate records of the killed civilians in the *županijas* (counties) directly impacted by conflict (Sekula Gibač 2015). We cross-referenced these with the research on overall demographic losses and people who sustained injuries in the same areas (Živić 2005a; 2005b; Benjak 2017). Furthermore, the maps of electoral units and *županijas* were cross-referenced with the official maps of the battlefields (Žunec 1998a; 1998b; Hrvatska enciklopedija 2020).

10 Table A1 in Online Appendix shows that the MPs from the larger parties are not necessarily asking more oral questions, Figure A2 illustrates the proportions, and Note A3 describes the formal procedures of Question Time in the Croatian parliament that support this observation. A7 presents the breakdown of the oral questions by the party of asking MP.

11 See Tables A4 and A5 in Online Appendix for the full breakdown of the questions’ areas.
