# Supplementary material for: Grandstanding Instead of Deliberative Policy-Making: Transitional Justice, Publicness and Parliamentary Questions in the Croatian Parliament
Source: J Interv Statebuild. 2024 Jul 1;18(5):598–619. doi: 10.1080/17502977.2024.2362001 (PMC11869769; doi:10.1080/17502977.2024.2362001)
Supplement: Supplementary Material [file RISB_A_2362001_SM4841.pdf]

Appendix. Grandstanding Instead of Deliberative Policy-Making:
Transitional Justice, Publicness and Parliamentary Questions in the Croatian Parliament

Content

A1. The Relationship between Party Size and Question Form (Oral/Written)
A2. The Procedure of Parliamentary Questions in Croatia
A3. Parties of Asking MP: Proportions of Oral and Written Questions
A4. Breakdown of "Other" Areas from Figure 4. Oral Questions
A5. Breakdown of "Other" Areas from Figure 4. Written Questions
A6. Model 3: Breakdown of the Coefficients
A7. Oral Questions: Breakdown by the Party of Asking MP

A1. The Relationship between Party Size and Question Form (Oral/Written).

Table A1 presents the estimates of the coefficients of dummy variables for the parties of MPs asking the PQs, while controlling for the parliamentary term. The dependent variable equals one if the question is oral. For simplified interpretation, the results of the Ordinary Least Squares (OLS) estimation are provided.

The estimates support the claim that party size is not necessarily directly linked to the choice between oral and written questions. As evidenced by the table, the estimate of the coefficient for the dummy variable for HDZ, the largest party in terms 5, 6, 8, and 9, is lower than the coefficients for some of the smaller parties (e.g. HRAST, Stranka Rada i Solidarnosti); the estimate of the coefficient for SDP, the second-largest party in terms 5, 6, 8, and 9, and the largest party in terms 4 and 8, is lower than the coefficient for dummy variables for most other parties. Similar tendency holds if individual-level controls and their combinations are included into the model (gender, age, co-partisanship, party ideology).

Table A1. DV: The Question is Oral.

Blok za Hrvatsku	0.06087 (0.41036)
DC	-0.69652*** (0.15914)
Demokrati	-0.93913*** (0.23425)
GLAS	-0.68913*** (0.14572)
HDS	-0.30755* (0.1581)
HDSSB	-0.77173*** (0.13256)
HDZ	**-0.34291*** (0.13036)**
HGS	-0.75*** (0.17156)
HNS	-0.85626*** (0.13099)
HRAST	-0.18913 (0.23425)
HRID	-0.4983 (0.30591)
Hrvatski laburisti-Stranka rada	-0.9727*** (0.13027)
HSD	-0.9461*** (0.20556)
HSLS	-0.45847*** (0.14484)
HSP	-0.70222*** (0.1353)

HSP AS	-0.93419*** (0.13715)
HSS	-0.83034*** (0.13153)
HSU	-0.62655*** (0.13508)
ID-DI	-0.83333*** (0.14786)
IDS	-0.83686*** (0.13224)
LIBRA	-0.81859*** (0.14981)
LS	-0.21145 (0.41037)
MDS	-1.12321*** (0.14659)
MOST	-0.83556*** (0.13175)
Neovisni za Hrvatsku	-0.05024 (0.18398)
Nezavisni	-0.74624*** (0.13118)
NLM	-0.84822*** (0.17547)
NLSP	-0.9983** (0.41142)
Novi Val	-0.98592*** (0.13766)
ORaH	-0.97692*** (0.1341)
PGS	-0.81145*** (0.16471)
Promijenimo Hrvatsku	-0.73913*** (0.16469)
Reformisti	-0.85706*** (0.15501)
SBHS	-0.21145 (0.41037)
SDAH	-0.51228*** (0.17535)
SDP	**-0.78018*** (0.13034)**
SDSS	-0.21282 (0.13767)
SIP	-0.88913*** (0.15684)
SMSH	-0.6058*** (0.18398)
SNAGA	-0.66641*** (0.17547)
Stranka rada i solidarnosti	-0.3509** (0.16104)
Živi zid	-0.91933*** (0.13167)
Term6	-0.09868*** (0.01403)
Term7	-0.21145*** (0.01478)
Term8	-0.21314*** (0.03297)
Term9	-0.27231*** (0.01421)
Intercept	1.21145*** (0.13052)
Rsq	0.278
Observations	9,141

Note: The dependent variable equals one if the question is oral. Standard errors in parentheses. ***p ≤0.001, **p ≤0.05, *p ≤0.01.

Party ranking in accordance with their size:

Term 5: HDZ, SDP, HNS
Term 6: HDZ, SDP, HSS
Term 7: SDP, HDZ, HNS
Term 8: HDZ, SDP, Most

Term 9: HDZ, SDP, Most

Note A2. The Procedure of Parliamentary Questions in Croatia.

The full procedure of PQs in Croatia in outlined in Poslovnik Hrvatskoga Sabora (*Rules of Procedure of the Croatian Parliament*).[1] The document includes the sections that state that:

- The priority is given to questions from independent MPs, representatives of national minorities not affiliated with parliamentary clubs, and MPs from political parties not belonging to parliamentary clubs. MPs' clubs can request to change the order of up to three questions if they consider certain questions to be more pertinent in the current situation (Article 135).
- The Speaker has the authority to determine the duration of discussions, thereby influencing the depth of deliberation on items listed in the agenda.

Figure A3. Parties of Asking MP: Proportions of Oral and Written Questions.

Figure 3 exemplifies the claim that the party size is not necessarily directly linked to the choice between oral and written question.

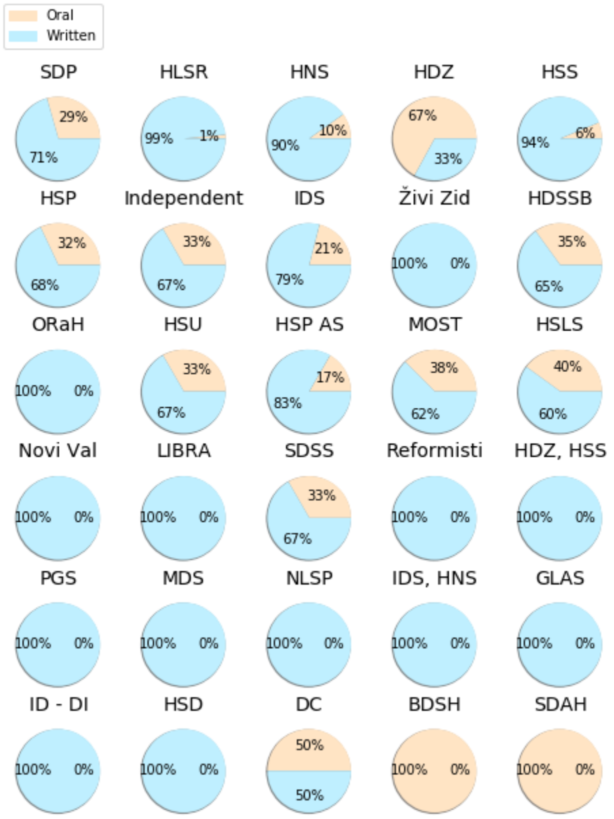

Note: The visualisation is based on all the parliamentary questions asked in the Croatian parliament (2004–2020).

[1] https://www.sabor.hr/sites/default/files/uploads/inline-files/Poslovnik%20Hrvatskoga%20sabora%20-%20procisceni%20tekst%202018.pdf

Note A4. Breakdown of "Other" Areas from Figure 4. Oral Questions.

Area	Proportion of questions
Internal affairs and national security	3.76%
Education and science	3.29%
Conversion and privatization	2.82%
Constitutional system	2.35%
Organisation of state power	2.35%
Defence	1.88%
Pension system	1.88%
Organization of the judiciary	1.41%
Culture and art	0.94%
Economy	0.94%
Employment relationships	0.94%
Health insurance	0.94%
International law	0.94%
Public finance	0.94%
Public information	0.94%
Roads and traffic	0.94%
Tax system	0.94%
Urban planning	0.94%
Agriculture	0.47%
Associations	0.47%
Chamber of commerce and other chambers	0.47%
Citizens' rights	0.47%
Civil servants	0.47%
Construction	0.47%
Corporate law	0.47%
Family law relations	0.47%
Financial services supervision	0.47%
Industry	0.47%
Military and civilian war invalids	0.47%
Nature and environmental protection	0.47%
Procurement and prices	0.47%
State property	0.47%

Note A5. Breakdown of "Other" Areas from Figure 4, Written Questions.

Area	Proportion of questions
Pension system	3.86%

Property law	3.86%
Education and science	3.55%
Family law relations	3.40%
National budget	3.40%
Agriculture	3.24%
Employment relationships	2.78%
State administration and local self-government	1.85%
State property	1.85%
Conversion and privatization	1.70%
Health care system	1.54%
Public finance	1.39%
Constitutional system	1.23%
Civil servants	1.08%
Defence	1.08%
Energy and natural resources	1.08%
Nature and environmental protection	1.08%
Organization of the judiciary	1.08%
Roads and traffic	1.08%
Administrative procedures and disputes	0.93%
Corporate law	0.93%
Culture and art	0.93%
Health insurance	0.93%
Tax system	0.93%
Associations	0.77%
Economy	0.77%
Organisation of state power	0.77%
Water and utilities	0.77%
Civil litigation	0.62%
Concessions	0.62%
Construction	0.62%
Internal affairs and national security	0.62%
International law	0.62%
Procurement and prices	0.62%
Industry	0.46%
Railway transportation and transport benefits	0.46%
Disaster protection	0.31%
Financial services supervisory	0.31%
Forestry	0.31%
Monetary intervention	0.31%
Obligations	0.31%
Antifascism	0.15%
Audit and control	0.15%
Chamber of commerce and other chambers	0.15%

Citizens' rights	0.15%
Craft	0.15%
Electoral system	0.15%
Employment and social issues	0.15%
Food	0.15%
Institutes	0.15%
Insurance of property and individuals	0.15%
Public information	0.15%
Urban planning	0.15%
Water transportation	0.15%

A6. Model 3: Breakdown of the Coefficients.

TJ (Transitional Justice)	**-0.716*****<br>(0.110)
Co-partisan	2.662***<br>(0.091)
Party ideology of asking MP	
Far right	0.725***<br>(0.175)
Right-of-centre	1.015***<br>(0.092)
Left-of-centre	0.178**<br>(0.080)
NA (independent)	0.815***<br>(0.131)
Controls	
Female asking MP	-0.449***<br>(0.067)
Addressed to a female member of government	0.066<br>(0.074)
Education asking MP	0.277***<br>(0.040)
Age of asking MP	0.026***<br>(0.003)
Term 6	-0.473***<br>(0.085)
Term 7	-1.696***<br>(0.081)
Term 8	-1.015***<br>(0.206)

Term 9	-1.605***
	(0.083)
Intercept	-0.493
	(0.450)
Observations	9,142
McFadden's pseudo-Rsq	0.2558

Note. The dependent variable equals one if the question is oral. Standard errors in parentheses. ***p ≤0.001, **p ≤0.05, *p ≤0.01.

As evidenced by Model 3, men are more likely to ask oral questions than women, a higher level of education of an MP who is asking a question is positively liked to the probability of asking an oral question, and older MPs are more likely to ask oral questions, holding all the other variables constant. For instance, the estimated probability of a female MP (50 years old, with higher education, and belonging to a centrist party) asking an oral question about TJ addressed to a co-partisan female member of the cabinet in term 9 is 0.41.

Table A7. Oral Questions: Breakdown by the Party of Asking MP.

Party of asking MP	Oral questions, count	TJ	Other topics
BDSH	9	2 (22%)	7 (78%)
Blok za Hrvatsku	1	0 (0%)	1 (100%)
DC	8	1 (13%)	7 (88%)
GLAS	9	0 (0%)	9 (100%)
HDS	12	0 (0%)	12 (100%)
HDSSB	54	7 (13%)	47 (87%)
**HDZ**	**1,027**	**96 (9%)**	**931 (91%)**
HGS	3	0 (0%)	3 (100%)
HNS	169	7 (4%)	162 (96%)
HRAST	3	1 (33%)	2 (67%)
HRID	1	0 (0%)	1 (100%)
Hrvatski laburisti-Stranka rada	29	0 (0%)	29 (100%)
HSD	1	0 (0%)	1 (100%)
HSLS	26	1 (4%)	25 (96%)
HSP	58	6 (10%)	52 (90%)
HSP AS	5	1 (20%)	4 (80%)
HSS	104	0 (0%)	104 (100%)
HSU	51	2 (4%)	49 (96%)
ID-DI	5	0 (0%)	5 (100%)
IDS	69	4 (6%)	65 (94%)
LIBRA	11	0 (0%)	11 (100%)
LS	1	0 (0%)	1 (100%)
MDS	3	0 (0%)	3 (100%)

MOST	48	2 (4%)	46 (96%)
Neovisni za Hrvatsku	8	0 (0%)	8 (100%)
Nezavisni	130	5 (4%)	125 (96%)
NLM	1	0 (0%)	1 (100%)
Novi Val	1	0 (0%)	1 (100%)
ORaH	3	0 (0%)	3 (100%)
PGS	6	0 (0%)	6 (100%)
Promijenimo Hrvatsku	3	0 (0%)	3 (100%)
Reformisti	3	0 (0%)	3 (100%)
SBHS	1	0 (0%)	1 (100%)
SDAH	7	1 (14%)	6 (86%)
**SDP**	**752**	**53 (7%)**	**699 (93%)**
SDSS	62	2 (3%)	60 (97%)
SIP	1	0 (0%)	1 (100%)
SMSH	3	0 (0%)	3 (100%)
SNAGA	3	0 (0%)	3 (100%)
Stranka rada i solidarnosti	10	0 (0%)	10 (100%)
Živi zid	10	0 (0%)	10 (100%)
